# Between algorithms and probes: evaluation of a digital tool’s performance in periodontitis classification

**DOI:** 10.1590/1807-3107bor-2026.vol40.086

**Published:** 2026-11-09

**Authors:** Bernardo Favini DALL’AGNOL, Isadora dos Santos ROTTA, Stephanie Anagnostopoulos FRIEDRICH, Gabrielle PEDRONI, Cassiano Kuchenbecker RÖSING, Juliano CAVAGNI

**Affiliations:** (a) Universidade Federal do Rio Grande do Sul – UFRGS, School of Dentistry, Department of Periodontology, Porto Alegre, RS, Brazil

**Keywords:** Periodontitis, Diagnosis, Reproducibility of Results, Decision Trees

## Abstract

This study evaluated the use of a digital tool as a complementary resource for classifying periodontitis patients by comparing its results with those from a standard clinical examination. A secondary analysis was performed using data from a cross-sectional observational study with 200 participants. Each underwent a full-mouth periodontal examination, and clinical diagnosis was established by two experienced dentists following the 2017 AAP/EFP Classification System. Radiographic data were not available. The same clinical data were entered into an online digital tool (PerioHelper), which uses a decision-tree model to provide an automated diagnosis according to the same classification criteria. Agreement between the clinical and digital diagnoses was assessed using Cohen’s Kappa coefficient for stage, grade, extent, and their combinations. Kappa values were 0.530 (95%CI: 0.442–0.618) for stage, 0.968 (95%CI: 0.933–1) for extent, and 0.920 (95%CI: 0.863–0.977) for grade. Combined analyses of stage and extent, and of stage, extent, and grade showed kappa values of 0.570 (95%CI: 0.494–0.646) and 0.580 (95%CI: 0.506–0.654), respectively. The Sankey diagram revealed that most discrepancies occurred at Stage II diagnosis, where just over 50% of the tool’s classifications matched those of the dentists. These results suggest that the digital tool provides support for periodontal diagnosis, particularly in defining extent and grade, though it has limitations in accurately identifying the disease stage, especially Stage II. It should be noted that this analysis was based exclusively on clinical parameters, without radiographic assessment, due to ethical and methodological considerations. This limitation may affect the precision of stage determination.

## Introduction

The 2017 Classification of Periodontal and Peri-Implant Diseases and Conditions proposed by the American Academy of Periodontology (AAP) and European Federation of Periodontology (EFP) marked a turning point in contemporary periodontology by introducing a structured framework for staging and grading periodontitis based on disease severity, complexity, extent, and risk of progression.^1-3^ Unlike the previous 1999 classification, which presented challenges in distinguishing between chronic and aggressive forms of the disease and lacked parameters for assessing progression or treatment stability, the updated model sought to align clinical diagnosis with current scientific knowledge and improve patient care over time.^1,2^

Despite its scientific robustness, the practical implementation of the 2017 classification has proven complex in routine clinical settings. Accurate diagnosis requires integrating a wide range of clinical and radiographic parameters with systemic and behavioral risk factors, a process influenced by clinician experience and the inherent subjectivity of clinical evaluation.^4^ This complexity has prompted the development of clinical decision support systems (CDSS) aimed at assisting dental professionals in applying diagnostic criteria more systematically and consistently.

Digital tools based on artificial intelligence (AI), artificial neural networks (ANN), and decision tree (DT) algorithms have gained increasing relevance in dentistry, particularly in diagnostic imaging, orthodontic assessments, and caries detection.^5,6^ In periodontology, however, the use of such tools remains limited, and their clinical validation is still incipient. Nonetheless, these systems show potential to enhance diagnostic accuracy, reduce inter-examiner variability, and optimize clinical workflows.^6^

Among these technologies, PerioHelper is a web-based digital platform developed to assist in the classification of periodontitis according to the 2017 framework. It operates using a decision tree logic that guides clinicians through a structured sequence of clinical inputs—including clinical attachment loss, probing depth, bone loss pattern, smoking habits, and diabetes status—ultimately generating an automated classification in terms of stage and grade. By standardizing data interpretation, PerioHelper aims to support clinical decision-making while minimizing diagnostic inconsistencies.

However, for tools like PerioHelper to be safely integrated into clinical practice, their diagnostic performance must be evaluated against conventional full-mouth periodontal examination, which remains the gold standard in periodontal diagnosis.^7^ Validation studies are crucial to determine their reliability, particularly in borderline or ambiguous cases, where clinical reasoning is essential and algorithmic tools may fall short.

Therefore, the aim of this study was to evaluate the diagnostic performance of the PerioHelper digital tool in the classification of periodontitis, by assessing its agreement with clinical diagnoses performed by experienced dentists, based on the 2017 Classification of Periodontal Diseases. The hypothesis of this study was that the digital tool (PerioHelper) performs significantly worse than conventional clinical examination in the classification of periodontitis. The goal is to test whether this difference is acceptable in terms of diagnostic agreement.

## Methods

### Study design

This study is a secondary analysis based on data derived from a cross-sectional observational clinical study.

### Ethical considerations

This study was conducted in accordance with the principles outlined in the Declaration of Helsinki and followed the ethical guidelines established by the Brazilian National Health Council, as stated in Resolution No. 466/12. The research protocol was reviewed and approved by the Research Ethics Committee of the Federal University of Rio Grande do Sul (UFRGS) under approval number 6.569.466.

### Sample composition and stratification

This secondary analysis was based on a sample of 200 individuals who participated in the primary cross-sectional observational study. Participants were recruited by convenience sampling through verbal invitations and distribution of printed flyers at the School of Dentistry of the Federal University of Rio Grande do Sul (UFRGS) and at the Instituto de Ensino, Aperfeiçoamento e Pesquisa do Mercosul (IEAPOM). All individuals completed a sociodemographic questionnaire and underwent intraoral examinations for the assessment of hard and soft oral tissues, including a comprehensive dental assessment and a full-mouth periodontal examination. Based on the clinical findings, each participant received a periodontal diagnosis established by calibrated examiners. All 200 individuals from the original sample were included in the present analysis. The sample of the primary study was structured to ensure demographic and systemic diversity, including at least 40% male participants, 20% smokers, 10% individuals with diabetes, and an approximately balanced distribution across three age categories (18–30, 31–50, and 51–70 years). These quotas were established a priori to guarantee representation of key variables known to influence periodontal status and to strengthen diagnostic agreement testing.

### Eligibility criteria

Participants included in the primary study were individuals aged between 18 and 70 years, with a minimum of 12 teeth present in the oral cavity, and who were available to participate in the study procedures.

Exclusion criteria comprised dental professionals, pregnant or breastfeeding women, and individuals who had undergone recent periodontal treatment, including scaling and root planing within the six months prior to the study or dental prophylaxis within the last three months. Participants presenting with acute oral conditions or requiring antimicrobial prophylaxis prior to dental procedures were also excluded.

### Clinical examination

In the periodontal clinical examination of the primary study, the following parameters were evaluated:

- Plaque Index (Visible Plaque Index – VPI)^8^: presence or absence of visible plaque on buccal and lingual surfaces after air-drying. Results are expressed as the percentage of sites with plaque.
- Gingival Index (Gingival Bleeding Index – GBI)^8^: presence or absence of bleeding within 10 s after gentle probing. Results are expressed as the percentage of sites with bleeding.
- Probing Depth (PD): measured in millimeters from the gingival margin to the most apical probed portion of the periodontal pocket or gingival sulcus.
- Bleeding on Probing (BOP): recorded dichotomously according to the presence or absence of bleeding during examination.
- Clinical Attachment Loss (CAL): evaluated as the distance, in millimeters, between the cementoenamel junction and the most apical probed portion of the periodontal pocket or gingival sulcus.

Probing depth analysis was conducted by examining all dental surfaces except for the distal surface of second molars. This exclusion was due to methodological and clinical reasons such as potential bias from increased probing depths, the presence of hyperplastic gingival flaps in cases of third molar eruption, pericoronitis, limited access and probing difficulty, and anatomical variations. Inclusion of this site may distort clinical results in research methodologies.^9^ The analysis of clinical attachment loss was performed following the periodontal diagnostic criteria established by the 2017 World Workshop Classification of Periodontal Diseases.^10^

### Reproducibility

Prior to data collection, examiner calibration was conducted to ensure consistency and reliability in periodontal measurements. Three examiners independently evaluated a subset of participants for PD and CAL measurements. Agreement with the gold-standard examiner, assessed using the Intraclass Correlation Coefficient (ICC), ranged from 0.90 to 0.94 for PD and from 0.85 to 0.88 for CAL.

In the present study, the reproducibility of periodontal diagnosis was also evaluated. The diagnosis of the 200 participants was performed by two dentists (ISR and SAF), with inter-examiner agreement assessed using Cohen’s kappa coefficient. Kappa values were interpreted according to a six-level scale: poor (<0.00), slight (0.00–0.20), fair (0.21–0.40), moderate (0.41–0.60), substantial (0.61–0.80), and almost perfect (0.81–1.00). The inter-examiner assessment resulted in a kappa value of 0.830 (almost perfect). Any discrepancies in the final diagnosis were resolved by a reference examiner (CKR).

### Data storage

The data obtained in the primary study, following the examination and diagnosis of all 200 individuals, were collected and recorded in an electronic document management system through the completion of standardized digital forms. These forms included mandatory fields related to personal information, findings from the examination of oral hard and soft tissues, a full dental examination, and a comprehensive periodontal clinical examination.

### Periodontal diagnosis

For periodontal diagnosis based on the clinical examination, a diagnostic flowchart was established following the studies by Holtfreter *et al*. and dos Anjos et al.^11,12^ Periodontitis was diagnosed by the presence of interproximal CAL ≥1 mm in at least two non-adjacent teeth or CAL ≥ 3 mm with PD > 3 mm in at least two teeth. Disease staging criteria were as follows: Stage I: CAL of 1 to 2 mm; Stage II: CAL of 3 to 4 mm and PD ≤6 mm in at least two non-adjacent teeth; Stage III: CAL of 3 to 4 mm and PD ≥ 6 mm in at least two non-adjacent teeth or CAL ≥ 5 mm with ≥ 20 remaining natural teeth; Stage IV: CAL ≥ 5 mm and < 20 remaining natural teeth.

Fewer than 20 remaining natural teeth was also considered when there were fewer than 10 natural teeth in one arch or fewer than 10 pairs in occlusion. To determine disease extent, the number of teeth affected at the severity level defined by the stage was used, categorized as localized (< 30%) or generalized (≥ 30%). For ethical reasons, full-mouth periapical radiographic surveys were not performed in all participants; therefore, radiographic examination was not considered in the present study.

For grading, the relative CAL at the most severely affected tooth was calculated, considering the affected root surface (mesial/distal), participant sex (female/male), and tooth type. Modifying factors for grading included smoking status (never smoker, former smoker, current smoker <10 cigarettes/day, current smoker ≥10 cigarettes/day) and self-reported diabetes diagnosis (no diabetes, controlled diabetes, uncontrolled diabetes). Smoking fewer than 10 cigarettes/day was classified as grade B; smoking 10 or more cigarettes/day as grade C. Controlled diabetes was considered grade B, whereas uncontrolled diabetes was considered grade C.

### Digital decision tree approach for periodontal disease diagnosis and classification

The digital tool used in this study was PerioHelper *– Periodontal Staging and Grading Online*. PerioHelper is a web-based digital platform developed in 2022 that employs a DT system to establish the diagnosis and classification of periodontal diseases. Its operational logic is based on the 2017 World Workshop Classification of Periodontal Diseases. PerioHelper guides the clinician through a logical sequence of branching questions and answers, leading to a single final outcome—core logic inherent to the DT model. The tool functions by inputting clinical data, including clinical attachment level, probing depth, percentage and pattern of bone loss, and relevant health behaviors previously obtained by the dental professional through clinical interview and physical examination, in order to automatically determine the Stage and Grade of periodontitis. For periodontal diagnosis, the tool uses the following clinical parameters:

#### Determination of periodontal disease stage

- Severity: The tool assesses disease severity based on the level of interproximal clinical attachment loss, categorized into: 1–2 mm, 3–4 mm, greater than 5 mm, or not available; and on the number of teeth lost due to periodontal disease, categorized as: no tooth loss, up to 4 teeth lost (≤4), or 5 or more teeth lost (≥5).
- Complexity: Case complexity is assessed based on probing depth, categorized as: ≤ 4 mm, ≤ 5 mm, or ≥ 6 mm; and bone loss pattern, classified by the tool as predominantly horizontal or with vertical bone loss ≥ 3 mm.
- Additional information: As additional parameters, the tool considers:
  - Furcation involvement, categorized as: absent or Grade I, Grade II, or Grade III involvement;
  - Presence of moderate ridge defects, recorded as “yes” or “no”;
  - Need for complex rehabilitation, which considers the following factors: masticatory dysfunction, secondary occlusal trauma, tooth mobility ≥Grade 2, presence of severe ridge defects, bite collapse, or fewer than 20 remaining teeth. This variable is also recorded as “yes” or “no”.
- Extent: Disease extent is assessed by the percentage of affected teeth and classified as localized (<30%) or generalized (≥30%).
- Distribution: Distribution is evaluated based on the molar/incisor pattern, recorded as either “yes” or “no”.

Determination of periodontal disease grade

- Primary Criteria: As primary grading criteria, the tool uses radiographic bone loss rate or clinical attachment loss progression, categorized as: none in the past 5 years; < 2 mm in the last 5 years; ≥2 mm in the last 5 years; or not available.
- Grade Modifiers: The tool incorporates smoking and diabetes status as grade modifiers. Smoking status is categorized as: non-smoker, smoker of <10 cigarettes/day, or smoker of ≥10 cigarettes/day. Diabetes status is categorized as: normoglycemic or no diagnosis of diabetes, diabetic with HbA1c <7%, or diabetic with HbA1c ≥7%.

Missing data in PerioHelper included the following variables: perio-caused tooth loss (staging), type of bone loss (staging), moderate ridge defects (staging), distribution (staging), and case phenotype (grading). Radiographic bone loss or CAL (grading) was consistently classified as not available. The bone loss/age ratio was recorded according to the following categories: <0.25, 0.25–1.0, and >1.0.

## Data analysis

Descriptive statistics were used to summarize the main characteristics of the primary study and included: means (standard deviations) for continuous variables (age, VPI, GBI, PD and CAL) and frequency distributions for categorical variables (gender, smoking status, diabetes status). For the descriptive analysis, smoking status was dichotomized into current or former smokers (former smoker, current smoker <10 cigarettes/day, and current smoker ≥10 cigarettes/day) and never-smokers. Diabetes status was dichotomized into diabetic (controlled and uncontrolled diabetes diagnoses) and non-diabetic. Normal distribution was tested using the Shapiro-Wilk test.

Agreement between the clinical diagnosis established by the dentist and the digital tool was assessed using Cohen’s Kappa coefficient with 95% confidence intervals (95% CIs). Periodontal diagnosis was treated as a categorical, non-ordinal variable. Analyses were performed according to stage, grade, and extent, as well as their combined classifications: stage and extent; stage, grade, and extent. Kappa values were interpreted according to Landis and Koch (1977). The percentage of complete agreement was also calculated.

## Results

Descriptive data of the sample are presented in Table 1. The sample consisted of 200 individuals, with a mean age of approximately 41 years and a predominance of females (57%). Regarding smoking status, 58.5% of participants were classified as current or former smokers. Among these, 39% were identified as current smoker (< 10 cigarettes/day or ≥10 cigarettes/day) (data not shown). Diabetes mellitus was reported by 13% of participants, including both controlled and uncontrolled cases.

**Table 1:** Descriptive characteristics of the primary study sample.

Variable		
Age (Mean ± SD)	41.2 ± 13.7	
	Category	Estimate n (%)
Gender	Male	86 (43)
	Female	114 (57)
Smoking status	Never-smoker	83 (41.5)
	Former smoker	39 (19.5)
	Current smoker (< 10 cigarettes/day)	46 (23)
	Current smoker (≥ 10 cigarettes/day)	32 (16)
Diabetes status	Normoglycemic/no diagnosis of diabetes	174 (87)
	Diabetic with HbA1c < 7%	24 (12)
	Diabetic with HbA1c ≥ 7%	2 (1)
	Mean ± SD	Min/Max
Visible Plaque Index (%)	63.3 ± 25.3	2.0/100.0
Gingival Bleeding Index (%)	36.7 ± 24.2	1.0/97.0
Probing Depth (mm)	2.1 ± 0.6	1.3/5.2
Clinical Attachment Loss (mm)	1.6 ± 1.1	0.1/6.9

As for clinical periodontal parameters, the observed means were 63.3 for the VPI and 36.7 for the GBI. The mean PD was 2.1 mm, and the mean CAL was 1.6 mm. All periodontal parameters showed a wide range of values between the observed minimum and maximum.

Table 2 presents the agreement indices between the diagnoses performed by the dentist and the digital tool, considering the criteria of stage, extent, and grade of periodontitis, as well as the combined analyses of stage and extent, and stage, extent, and grade. Moderate agreement was observed for the diagnosis of periodontitis stage, with a Kappa value of 0.530 and an agreement rate of 66%. In contrast, the assessment of disease extent showed excellent agreement, with a Kappa of 0.968 and an agreement rate of 99%. Similar results were observed for disease grade, with a Kappa of 0.920 and an agreement rate of 97%. In the combined analyses—stage and extent, and stage, extent, and grade—the agreement was classified as moderate with Kappa values of 0.570 and 0.580, and agreement rates of 66% and 64%, respectively.

**Table 2:** Results of the analysis using Cohen’s Kappa coefficient (95% confidence interval), considering the following parameters: stage, grade, extent, and the combination of variables. (n = 200)

Dentist vs. Digital Tool		
Variable	Agreement (%)	Kappa (CI)
Stage	66	0.530 (0.442– 0.618)
Grade	97	0.920 (0.863–0.977)
Extent	99	0.968 (0.933–1)
Stage and extent	66	0.570 (0.494–0.646)
Stage, grade, and extent	64	0.580 (0.506–0.654)

CI: confidence interval.

In addition, the agreement analysis between the periodontitis stage diagnosis made by the digital tool and that made by the dentist is illustrated in the Sankey diagram shown in Figure. This figure represents the discrepancies between the diagnoses performed by the dentist and those generated by the digital tool. It is clearly observable that most of the discrepancies occurred in cases of Stage II periodontitis. Just over 50% of the cases diagnosed as Stage II by the digital tool were classified in the same way by the dentist.

**Figure:**
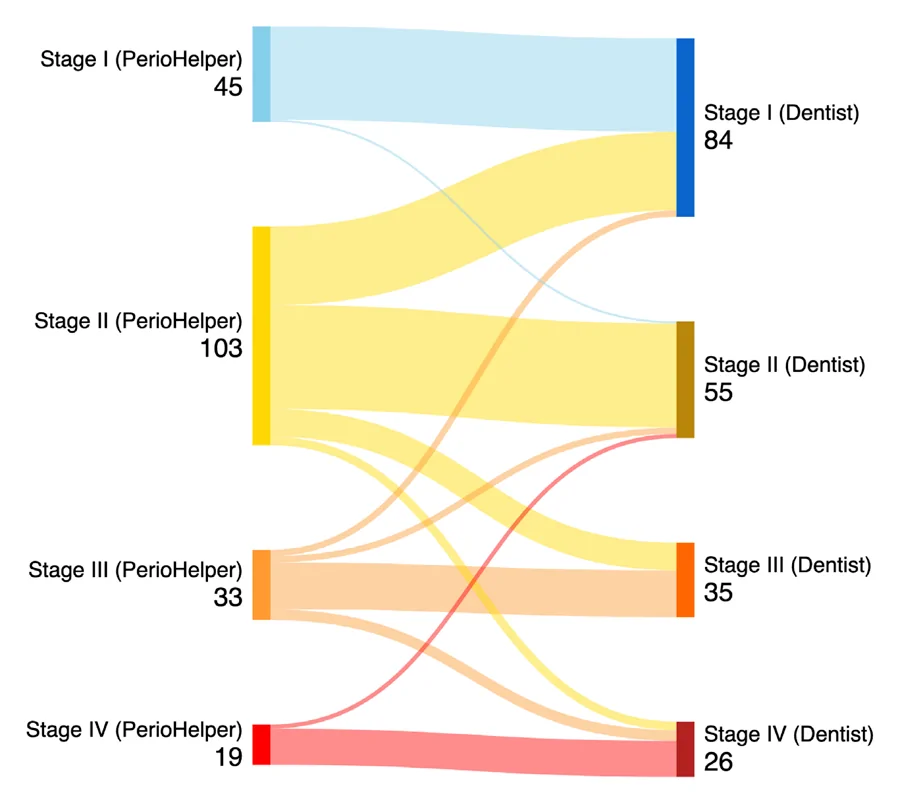
Sankey diagram used to compare periodontitis stage classification between diagnoses made by PerioHelper and the dentist.

## Discussion

The present study aimed to assess the performance of a digital tool (PerioHelper) in assisting the classification of periodontitis, by comparing its diagnostic output with that of the conventional clinical method performed by an experienced dentist. The findings suggest that PerioHelper can be used as a supportive tool for periodontitis classification, particularly regarding the assessment of extent and grade, for which excellent agreement was observed with the clinical diagnosis. In contrast, the tool showed only moderate accuracy in determining the stage of periodontitis, with most of the discrepancies concentrated in Stage II cases.

The conventional periodontal clinical examination, performed by dentists in the primary study, involved the analysis of various parameters, such as probing depth, clinical attachment loss, bleeding on probing, presence of biofilm, and number of natural teeth. It is important to highlight that although the use of technologies like PerioHelper represents an advancement in Periodontology, the complete periodontal clinical examination performed by a professional remains the gold standard for periodontal diagnosis.^14^ This is because the diagnostic process requires training and clinical judgment, along with individualized interpretation, which enables the detection of nuances often beyond the scope of digital tools.

In this study, a moderate agreement of 64% was found between the diagnosis provided by the digital tool and that made by the dentist when considering the stage, grade, and extent of periodontitis combined. This finding aligns with results from Marini *et al*., who assessed diagnostic accuracy between dentists and a digital application developed by the Italian Society of Periodontology and Implantology for staging and grading periodontitis.^15^ That study showed that only 53.6% of cases diagnosed by the app were correctly classified with respect to all criteria (stage, grade, and extent).

When assessed individually, the staging diagnosis provided by the digital tool in the present study also showed moderate accuracy, with a Cohen’s Kappa coefficient of 0.530. However, when compared to the findings by Marini *et al*., a notable difference is observed.^15^ In their study, aided by the digital application, dentists achieved near-perfect agreement levels, with a Kappa value of 0.818 for disease staging. This discrepancy may be explained by differences in data availability: in addition to data used in the present study (clinical attachment loss and probing depth), Marini *et al*. also included variables such as the number of teeth lost due to periodontitis, furcation involvement, and full-mouth periapical radiographs.^15^ Furthermore, it is possible that the app used by Marini *et al*. employed a different decision-support system than a decision tree (as used by PerioHelper).^15^

Nevertheless, it is worth noting that in Marini *et al*. study, diagnostic accuracy for staging was significantly higher for the more severe forms of the disease. This is consistent with the current study’s findings. The Sankey diagram revealed that the greatest agreement between the digital tool and the dentist was in Stage IV, with 17 of 19 cases being concordant. In contrast, the largest diagnostic discrepancies occurred in Stage II, where among 103 cases identified by PerioHelper as Stage II, 40 were considered Stage I by the dentist. This suggests that both the digital tool evaluated in this study and the app used by Marini *et al*. tend to overestimate milder cases of the disease, which may be related to the so-called “gray zones” of classification.^15^ These zones refer to borderline or ambiguous cases defined by imprecise criteria, requiring more interpretive and contextual clinical analysis—something that tools based solely on logical decision-making, such as decision trees, may not perform with the same precision as a clinician.^4^ This reinforces once again the rationale for considering clinical periodontal examination combined with professional judgment as the gold standard in periodontal diagnosis.

In this context, digital decision-support tools such as PerioHelper may help mitigate diagnostic variability at the Stage II–III boundary by reinforcing a structured and criteria-based diagnostic workflow. By requiring the systematic input of key clinical parameters and integrating them simultaneously according to the 2017 AAP/EFP classification rules, the tool may assist clinicians in consistently verifying whether all staging thresholds are met before final stage attribution. In addition, the consolidation of multiple parameters into a single decision support output may reduce reliance on isolated clinical findings, which is particularly relevant in borderline cases.

When analyzed individually, the diagnosis of grade and extent of periodontitis using the digital tool demonstrated high reliability in distinguishing between localized and generalized forms and among Grades A, B, and C. Agreement for these variables was 99% and 97%, respectively. These results are consistent with findings from Parsegian *et al*., who also evaluated a decision tree-based digital tool, PocketPerio, grounded in the 2017 World Workshop Classification of Periodontal Diseases, similar to PerioHelper.^16^ According to their study, the use of the digital tool improved the diagnostic accuracy of periodontitis compared to conventional clinical assessment, allowing precise differentiation of patterns in disease extent and grade.

This high level of agreement between the digital tool and the dentist in determining grade and extent may be due to the nature and availability of data used in the evaluation. For example, the extent criterion is relatively more objective and standardized, requiring only the number of affected sites relative to the total, which is then converted into a percentage and input into the tool. As a result, good performance in this parameter may mask deficiencies in the system’s ability to qualitatively assess clinical data. In terms of grade, the high agreement could be attributed to the availability of both primary grading data, such as the rate of clinical attachment loss over the past five years, and modifying factors like smoking habits and systemic diseases such as diabetes. This finding highlights the digital tool’s strong dependence on comprehensive and accurate clinical data input to achieve correct diagnoses.

Regarding the study sample, the results showed a mean age of approximately 41 years, indicating a higher prevalence of periodontitis among adults in this age group, likely due to cumulative exposure to risk factors over time. The sample showed a predominance of female participants. In terms of smoking, 39% reported being current smokers, and 13% of participants had diabetes. It is well established that both smoking and diabetes are recognized risk factors for periodontitis, as both negatively influence the host’s inflammatory response and tissue healing. Furthermore, diabetes, especially when uncontrolled, may impair the response to periodontal treatment.^17^ The presence of these variables may have influenced the variability in clinical scenarios diagnosed by the tool, reinforcing the importance of including these factors in the system calibration process to improve diagnostic accuracy in real-world populations.

Therefore, the consistency between the findings of Marini *et al*. and Parsegian *et al*. highlights the potential effectiveness of the evaluated digital tool as a diagnostic aid in periodontitis.^15,16^ However, it is crucial to acknowledge that PerioHelper still requires refinement. Despite high agreement for extent and grade, the tool demonstrated lower precision in staging, potentially indicating limitations in the algorithm’s ability to simultaneously consider clinical variables and individualized patient data. Thus, relying solely on these tools—without critical analysis by a clinician—may result in diagnostic errors and inappropriate treatment planning. These findings emphasize the need for continued calibration of PerioHelper and its developers and call for more robust clinical validation studies to further explore the tool’s performance. It is important to emphasize that, at the time of this research, no prior validation studies of PerioHelper had been published. The present study therefore provides the first clinical evidence of its diagnostic performance. Future investigations should aim to replicate and expand these findings in larger and radiographically documented populations to establish external validity.

This study presents some limitations that should be considered when interpreting its findings. The main limitation concerns the absence of radiographic assessment, as no radiographs were obtained for any participant in the primary cross-sectional study. This decision followed the approved research protocol, which excluded radiographic exposure because of ethical and logistical reasons, given that the study involved healthy volunteers and was not intended for diagnostic or therapeutic purposes. Consequently, both the clinical and digital diagnoses relied solely on clinical parameters. This methodological choice restricts the differentiation between shallow pockets with bone loss and deep pockets without significant bone loss and may have affected the accuracy of stage classification. Moreover, the absence of radiographs limits the generalizability of these results, as radiographic evaluation is a suggested component of the 2017 AAP/EFP Classification. Future validation studies including radiographic datasets are therefore warranted to comprehensively assess and refine the performance of the PerioHelper algorithm.

The present study’s convenience sample limits population representativeness and statistical power, and the findings should be interpreted accordingly. The diagnostic variability observed between Stages II and III likely reflects the sensitivity of the 2017 AAP/EFP thresholds rather than clinically meaningful differences. Such borderline reclassification is an expected nuance of the diagnostic process and rarely changes therapeutic decisions.

The findings of this study highlight the growing potential of digital decision-support tools to improve diagnostic consistency in periodontitis classification. The moderate-to-substantial agreement observed between clinical and digital assessments suggests that structured algorithms, such as PerioHelper, can assist clinicians in systematically applying the 2017 AAP/EFP criteria and reducing subjectivity in borderline cases. This is particularly relevant in educational and large-scale clinical settings, where diagnostic reproducibility is essential for accurate case definition and treatment planning. However, the observed discrepancies—especially between Stages II and III—also emphasize that digital outputs should complement, rather than replace, the clinician’s judgment. Clinical expertise remains critical for integrating radiographic, anatomical, and patient-specific factors that may not be fully captured by algorithmic classification. As digital systems continue to evolve, their integration into clinical workflows may enhance diagnostic standardization, facilitate multicenter research comparability, and support evidence-based periodontal care.

## Conclusion

The digital tool demonstrated excellent performance in classifying the extent and grade of periodontitis, along with moderate agreement in defining the disease stage. These results indicate that the digital tool holds promise as a diagnostic aid in periodontal assessment, particularly regarding extent and grade. However, it presents limitations in staging—especially in Stage II—which highlight the need for further refinement and calibration to improve diagnostic accuracy.

## Data Availability

The data analyzed in this study are part of a larger clinical research database that has not yet been fully published. Therefore, the underlying dataset cannot be made publicly available at this time.
